# Role of the Appendicular Skeletal Muscle Index for Predicting the Recurrence-Free Survival of Head and Neck Cancer

**DOI:** 10.3390/diagnostics11020309

**Published:** 2021-02-14

**Authors:** Kun-Yun Yeh, Hang Huong Ling, Shu-Hang Ng, Cheng-Hsu Wang, Pei-Hung Chang, Wen-Chi Chou, Fang-Ping Chen, Yu-Ching Lin

**Affiliations:** 1 Division of Hemato-oncology, Department of Internal Medicine, Chang Gung Memorial Hospital, College of Medicine, Keelung & Chang Gung University, Keelung 222, Taiwan; yehtyng@gmail.com (K.-Y.Y.); xianfang87@gmail.com (H.H.L.); chw0098@gmail.com (C.-H.W.); ph555chang@gmail.com (P.-H.C.); 2 Department of Medical Imaging and Intervention, Chang Gung Memorial Hospital, Linkou & Chang Gung University, Taoyuan City 333, Taiwan; shuhangng@gmail.com; 3 Division of Hemato-oncology, Department of Internal Medicine, Chang Gung Memorial Hospital, College of Medicine, Linkou & Chang Gung University, Taoyuan City 333, Taiwan; wenchi3992@yahoo.com.tw; 4 Department of Obstetrics and Gynecology, Chang Gung Memorial Hospital, College of Medicine, Keelung & Chang Gung University, Keelung 222, Taiwan; fangping@cgmh.org.tw; 5 Healthy Aging Research Center, Chang Gung University, Taoyuan City 333, Taiwan; 6 Osteoporosis Prevention and Treatment Center, Chang Gung Memorial Hospital, Keelung 222, Taiwan; 7 Department of Medical Imaging and Intervention, Chang Gung Memorial Hospital, College of Medicine, Keelung & Chang Gung University, Keelung 222, Taiwan

**Keywords:** appendicular skeletal muscle index, head and neck cancer, dual-energy X-ray absorptiometry, recurrence-free survival rate, lean mass, concurrent chemoradiotherapy

## Abstract

Background: This study investigates whether the appendicular skeletal muscle index (ASMI) was an independent prognostic predictor for patients with locally advanced head and neck cancer (LAHNC) receiving concurrent chemoradiotherapy (CCRT) and whether there were any differences in lean mass loss in different body regions during CCRT. Methods: In this prospective study, we analyzed the clinicopathological variables and the total body composition data before and after treatment. The factors associated with the 2-year recurrence-free survival rate (RFSR) were analyzed via logistic regression analysis. Results: A total of 98 patients were eligible for analysis. The body weight, body mass index, and all parameters of body composition significantly decreased after CCRT. The pretreatment ASMI was the only independent prognostic factor for predicting the 2-year RFSR (hazard ratio, 0.235; 95% confidence interval, 0.062–0.885; *p* = 0.030). There was at least 5% reduction in total lean and fat mass (*p* < 0.001); however, the highest lean mass loss was observed in the arms (9.5%), followed by the legs (7.2%), hips (7.1%), waist (4.7%), and trunk (3.6%). Conclusions: The pretreatment ASMI was the only independent prognostic predictor for the 2-year RFSR of LAHNC patients undergoing CCRT. Asynchronous loss of lean mass may be observed in different body parts after CCRT.

## 1. Introduction

The total body composition represents the entire body mass primarily composed of lean mass, fat mass, and bone mass. Changes in the total body composition are common in patients with cancer in response to aging, metabolic demand, physiological alteration, and therapy. Accordingly, monitoring the fluctuation in the total body composition—instead of the changes in body weight (BW) or body mass index (BMI)—provides a more precise nutritional assessment during treatment [1]. Dual-energy X-ray absorptiometry (DXA) is a non-invasive clinical imaging method that has become the standard tool to evaluate lean mass, fat mass, and bone mineral density of the total body and different regions because it can precisely quantify each parameter with low radiation and costs [2,3,4].

The appendicular skeletal muscle index (ASMI), i.e., the sum of the lean muscle mass of the upper and lower extremities adjusted with height, is commonly used during DXA to diagnose whether elderly patients had a low lean mass or sarcopenia [5,6]. A low ASMI was significantly associated with the survival of patients with various cancers, such as biliary tract, breast, gastrointestinal, and lung cancers [7,8,9]. DXA has been used for assessing the total body composition change in patients with head and neck cancer treated with concurrent chemoradiotherapy (CCRT) [10,11,12,13,14]. However, no studies using DXA have evaluated the relationship between the ASMI and the 2-years recurrence-free survival rate (RFSR) in patients with locally advanced head and neck cancer (LAHNC) as well as the temporal changes in lean mass in different body regions after CCRT. We hypothesized that the ASMI may be an independent predictor for patients with LAHNC receiving CCRT while the effect of treatment toxicity on the lean mass of different body regions may be different, and that this asynchronous loss of lean mass may contribute to the prognostic role of the ASMI.

Accordingly, to resolve this uncertain issue and offset the bias effects from a heterogeneous population and different treatment protocols, we conducted a prospective cohort observational study of patients with locally advanced LAHNC who received standard CCRT and supportive care at a single institution. This study aimed to investigate whether the ASMI was an independent prognostic predictor for patients with LAHNC receiving CCRT and whether there were any differences in lean mass loss in different body regions during CCRT.

## 2. Materials and Methods

This prospective cohort study was performed between February 2015 and November 2017. This study was approved by the institutional review board (IRB) of the Chang Gung Memorial Hospital, Taiwan (IRB approval number: 103-3365A3) and was performed in accordance with the good clinical practice guidelines and the Declaration of Helsinki. Written informed consent was obtained from all patients. This trial was registered at ClinicalTrials.gov (identifier: NCT02854735).

### 2.1. Enrollment

Eligible patients were aged 20–75 years with histologically proven locally advanced head and neck squamous cell carcinoma. The patient population included a variety of tumors originating from the oral cavity, oropharynx, hypopharynx, and larynx, as well as metastatic cervical squamous cell carcinoma from unknown primary sites. The disease was staged according to the 7th edition of the American Joint Committee on Cancer (AJCC) staging system, which included stages III (T1-2, N1 or T3, N0-1), IVA (T4a, N0-1 or T1-4a, N2), and IVB (any T, N3 or T4b, any N). All the eligible patients had an Eastern Cooperative Oncology Group (ECOG) performance status score of ≤2 with adequate hematopoietic or organ function and could undergo CCRT. Patients were excluded if they had major gastrointestinal disorders, autoimmune disorders, end-stage renal failure, liver cirrhosis with intractable ascites, heart failure with New York Heart Association Classification IV, uncontrolled diabetes mellitus, or ongoing infections, or if they were receiving regular medications that could substantially modulate metabolism or weight, such as steroids or megestrol acetate.

### 2.2. CCRT Schedule

Curative CCRT alone was performed for patients with unresectable disease for the purpose of organ preservation. Adjuvant CCRT was performed for patients after surgery if they had (1) one of the two major risk factors of extranodal extension or a positive surgical margin; or (2) at least three of the following minor risk factors: pT4, pN1, close margin ≤4 mm, poor differentiation, perineural invasion, vascular invasion, lymph node invasion, or depth ≥10 mm. During CCRT, radiotherapy was delivered at a dose of 64–72 Gy in 32–36 fractions over an 8-week period, and concurrent chemotherapy with cisplatin (40 mg/m^2^) was administered once weekly. According to the treatment recommendations formulated by the head and neck cancer group in our institute, patients were eligible for this study even if they had received neoadjuvant chemotherapy before starting CCRT. Neoadjuvant chemotherapy was performed with docetaxel (60–75 mg/m^2^) on day 1, cisplatin (60–75 mg/m^2^) on day 1, and continuous infusion of 5-fluorouracil (1000 mg/m^2^/day) on days 1–4 every 3 weeks.

All patients received routine hydration, antiemetic medications, and adequate supportive care. In addition, all the patients were routinely referred to an early and intensive nutrition support program established in 2007 in our institute that included biweekly dietitian visits, mandatory feeding tube placement if the body weight loss (BWL) was >5% during the treatment course, timely caloric supplementation, and blood transfusion as needed [15]. More than 90% of the patients were admitted to the hospital for completion of the treatment course and had government healthcare support via the Taiwan National Health Insurance Program.

### 2.3. Clinicopathological Data and Nutrition Assessment Methods

Clinicopathological data were collected, including age, sex, body height, BW, ECOG performance status, comorbid diseases, tumor location, AJCC 7th edition of tumor node metastasis (TNM) stage, treatment modality (including the chemotherapy and radiotherapy doses), and history of smoking, alcohol consumption, and betel nut consumption. The severity of comorbid diseases was scored using the head and neck Charlson Comorbidity Index (HN-CCI), which was used to assess the presence of heart failure, pulmonary disease, cerebrovascular disease, peptic ulcers, liver disease, and diabetes [16]. Participants were considered smokers if they currently smoked tobacco or had smoked in the past. Participants were considered alcohol drinkers if they reported consuming alcohol ≥4 times per week. Participants were considered betel nut users if they reported taking this substance during the previous year.

The nutritional assessments were performed using BMI, BWL, and patient-generated subjective global assessment (PG-SGA) scores. BMI was defined as the weight in kilograms divided by the height in square meters (kg/m^2^); the BWL was calculated as (BW at diagnosis−ideal BW)/ideal BW × 100%. The PG-SGA scores ranged between 0 and 35, with scores of 0–3 indicating well nourished, 4–8 indicating moderately malnourished, and ≥9 indicating severely malnourished [17].

### 2.4. Body Composition Measurement

The total body composition was measured using dual-energy fan-beam X-ray absorptiometry (Lunar iDXA, GE Medical System, Madison, WI, USA). The scan mode (standard, thin, or thick) was selected automatically by the scanner software according to body size and BMI. Scans were analyzed using enCORE Software, version 15 (GE Lunar). All participants wore light indoor clothing and removed shoes and all items that may interfere with DXA results. Each participant was positioned according to the guidelines set by the International Society for Clinical Densitometry [18]. DXA was used to acquire the following parameters: total lean mass, total fat mass, and the ASMI. The total lean mass and total fat mass were normalized by dividing these parameters by height in square meters (kg/m^2^), and the total lean mass index (TLMI) and total fat mass index (TFMI) were calculated for analysis. The temporal changes in the lean mass after CCRT were examined in five different anatomical regions including the arms, legs, trunk, waist, and pelvis. The trunk region included the neck, chest, and abdominal and pelvic areas

All nutritional assessments and DXA studies were completed 1 week prior to initiating CCRT and within 1 week after CCRT was completed. The 2-year RFSR was defined as the proportion of patients who had no evidence of recurrence of the primary tumor within 730 days after CCRT initiation, which was used as the reference date owing to variation in the time for stage workups.

### 2.5. Statistical Analysis

The statistical analyses were performed using SPSS version 22.0 (SPSS Inc., Chicago, IL, USA). We assumed that the 2-year RFSR was 60% with a power of 80% and α error of 0.05; the minimum sample size was calculated to be 109. If the attrition rate was 10%, then the total number of patients that needed to be recruited was 120. Receiver operating characteristic (ROC) curves were used to determine the optimal cutoff values for the TLMI, TFMI, and ASMI. The associations among pretreatment clinicopathological data, nutritional assessments, DXA parameters, and the 2-year RFSR were analyzed by using logistic regression analysis with predefined cutoff values. The forward stepwise selection method was used for univariate and multivariate analyses for different nutritional parameters. All the independent variables that were significantly associated with the 2-year RFSR (*p* < 0.05) on univariate analysis were included in the multivariate analysis.

We investigated changes in body composition during CCRT to understand the impact of CCRT on the body composition of different body regions. The patients were classified into the high ASMI group (greater and equal to the cutoff value) and the low ASMI group (lower than the cutoff value) to investigate the effect of CCRT on the lean mass of different body regions, considering the two different health statuses of patients with LAHNC. The paired t-test was used to compare the body composition changes during CCRT. 

## 3. Results

**Figure 1:**
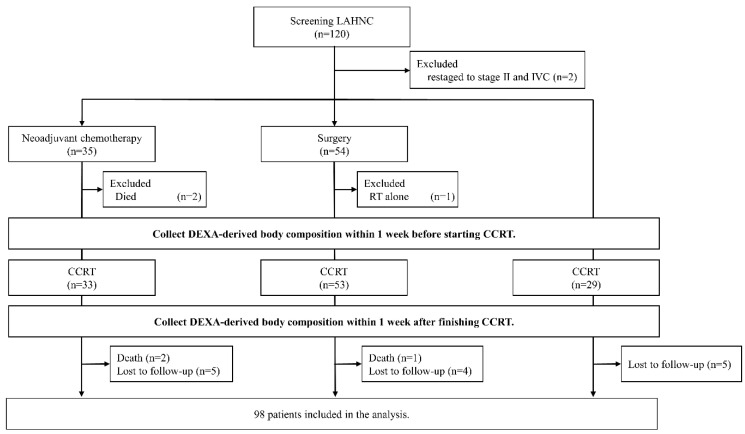
CONSORT diagram. Patients were considered to have completed planned therapy if they received at least 4 cycles of weekly cisplatin at 40 mg/m^2^ concomitant with radiotherapy (64–72 Gy). Lost to follow-up indicated patients who did not complete the Dual-energy X-ray absorptiometry (DXA) examination as scheduled. HNC, head and neck cancer; RT, radiotherapy; DXA, dual-energy X-ray absorptiometry; CCRT, concurrent chemoradiotherapy.

A total of 120 Chinese patients with LAHNC were recruited, 98 of whom were eligible for analysis in this study. The patient enrollment, allocation, treatment modality, and data collection details are presented in a CONSORT diagram (Figure 1). The baseline patient characteristics are summarized in Table 1. Among the 98 patients, male patients were predominant (96.9%) and the mean age was 53.4 years. The oral cavity was the most common tumor site (51.0%), followed by oropharynx (22.4%) and hypopharynx (18.4%). A high proportion of patients reported smoking (93.9%), alcohol consumption (77.6%), and betel nut consumption (65.3%). As for CCRT, 23 (23.5%) underwent CCRT alone, 49 (50.0%) received CCRT after curative surgery, and 26 (26.5%) underwent neoadjuvant chemotherapy followed by CCRT. At the 2-year follow-up after CCRT completion, 56 patients (57.1%) remained tumor-free.

Univariate analysis revealed that the tumor location, BWL > 10% at diagnosis, BMI, and the three pre-CCRT DXA parameters of the TLMI, TFMI, and ASMI were associated with the 2-year RFSR (Table 2). After adjusted for the location, BMI and TFMI in the multivariate logistic regression model, only the pretreatment ASMI (hazard ratio, 0.235; 95% confidence interval, 0.062–0.885; *p* = 0.030) was an independent prognostic factor for the prediction of the 2-year RFSR in patients with LAHNC (Table 2). 

**Figure 2:**
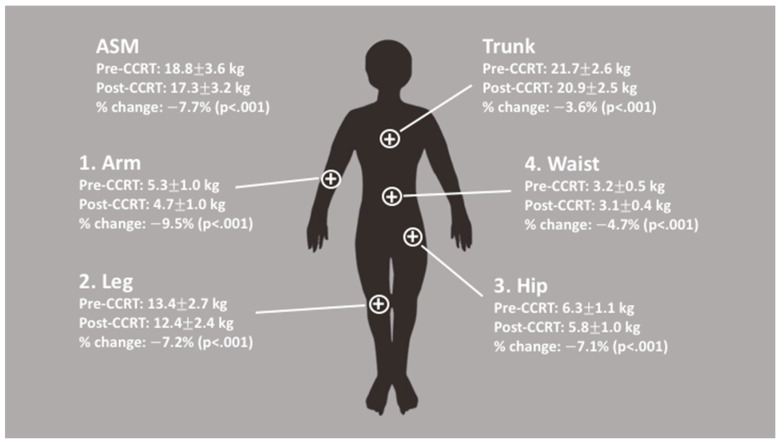
Details on the lean mass loss during CCRT. There was an asynchronous loss of lean mass in different body regions, with the highest loss of lean mass during CCRT observed in the arms, followed by the legs, hips, waist, and trunk. This suggests that CCRT had a higher impact on the lean mass of peripheral extremities (i.e., the appendicular skeletal muscle [ASM]) than on the central region (trunk) of the body.

**Figure 3:**
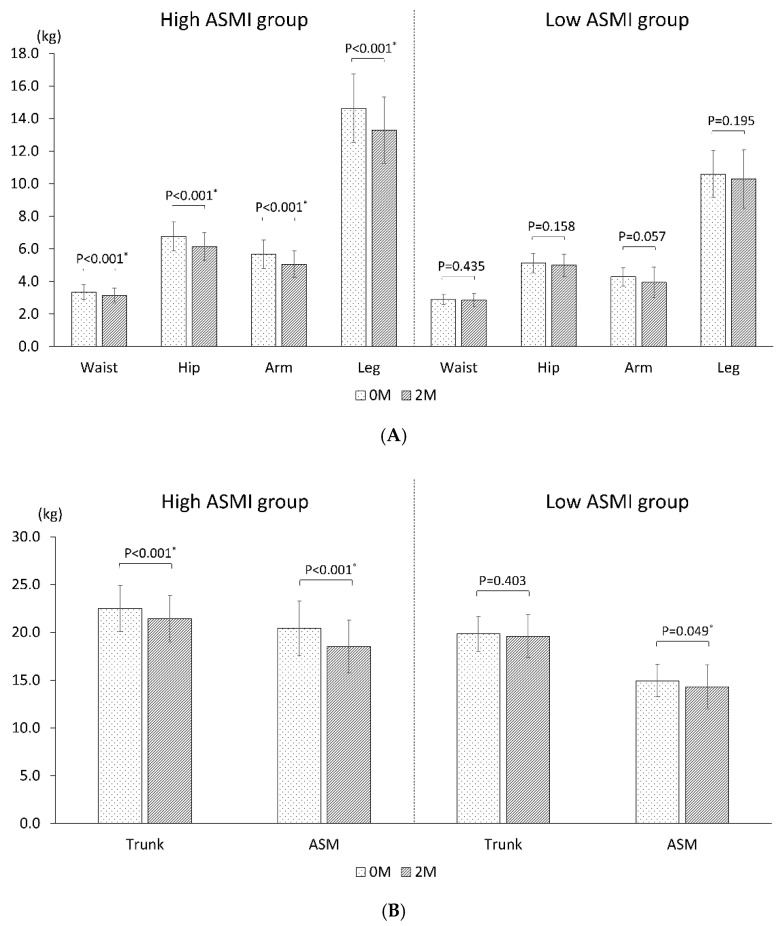
Comparison of lean mass changes during CCRT in patients with a high ASMI and in those with a low ASMI: (**A**) In different body regions; (**B**) In the central region (trunk) and peripheral extremities (ASM). In patients with a high ASMI, a significant loss of lean mass was observed consistently throughout different body regions. However, in patients with a low ASMI, there was no significant loss of lean mass in any region of the body; nevertheless, there was a significant loss of lean mass in the ASM mass. Significant *p* value were marked as *.

There were significant decreases in the BMI and all DXA parameters of body composition, i.e., the total lean mass, total fat mass, and ASMI after CCRT (Table 3), and there was an at least 5% reduction in all DXA parameters of body composition. A considerable discrepancy in the degree of lean mass loss was also observed in different body regions. The arms were found to have the highest loss of lean mass (9.5%), followed by the legs (7.2%), hips (7.1%), waist (4.7%), and trunk (3.6%) (Figure 2). This asynchronous loss of lean mass in different body regions resulted in a greater lean mass loss in the peripheral extremities (ASMI loss, 7.7%) than in the trunk or central region of the body. Moreover, the changes in lean mass showed different behaviors based on the health status of the patients with LAHNC. Among patients with a high ASMI, a significant loss of lean mass was found consistently throughout different body regions (Figure 3, Table S1). However, among patients with a low ASMI, there was no significant loss of lean mass in any region of the body, with a borderline significant loss (*p* = 0.049) of lean mass in the appendicular skeletal muscle (Figure 3, Table S2).

## 4. Discussion

To the best of our knowledge, this is the first study to evaluate the impact of body composition on the survival of patients with LAHNC undergoing CCRT using DXA. The pretreatment ASMI was the only independent prognostic factor that predicted the 2-year RFSR in patients with LAHNC. Although the total lean mass, total fat mass, and ASMI decreased significantly, with a decrease of at least 5% observed 1 week after CCRT, an asynchronous degree of lean mass loss was found in different body regions. This implied that CCRT had a greater influence on the lean mass of peripheral extremities (ASMI loss, 7.7%) than on the central body region (trunk loss, 3.6%), indicating that subsequently, patients with a low ASMI might have a lower tolerance to CCRT, thereby resulting in a lower 2-year RFSR.

In this study, there was a significant decline in all body composition parameters (the total lean mass, total fat mass, and ASMI) during CCRT; however, the ASMI was the only one nutritional index that was able to independently predict the 2-year RFSR in patients with LAHNC. This result was comparable to that observed for other advanced-stage cancers, as described in the introduction. There are three possible reasons why the ASMI was the only predictive factor. First, the ASMI was closely associated with ambulatory activity and physical functioning of humans [8]. Patients with a low ASMI are often weak and bedridden and more likely to experience complications such as pneumonia or infection [19]; hence, they often could not complete or tolerate the full treatment course of CCRT, thereby resulting in tumor recurrence. Second, the impact of CCRT on different body regions was different. In this study, the values of all body composition parameters decreased but in an asynchronous amount (Table 3 and Figure 2). The arms were found to have the highest loss of lean mass, followed by the legs, hips, waist, and trunk. As the indexes of the arms and legs constituted the ASMI, the predominant loss of lean mass was found in the ASMI. Our results were comparable to those obtained in the study by Fouladiun et al. [1]. They used DXA to assess the total body composition of 311 patients with stage IV cancer, most of them with gastrointestinal and hepatobiliary tract tumors. They found that the lean mass was lost preferentially from the arms and extremities, whereas the trunk tissue gained relative weight. Third, the ASMI could represent the status of the energy reservoir of the entire body. In fact, patients with head and neck cancer commonly experienced an energy deficit of more than 55,000 total calories during CCRT [20]. This energy deficiency could be related to insufficient dietary intake and metabolic derangement because of inflammation and anti-cancer treatment [2]. The evidence of ASMI represents the status of energy reservoir of the entire body can be shown by this study from stratified patients into high and low ASMI groups. In patients with a high ASMI, a significant loss of lean mass was found throughout different body regions; however, in patients with a low ASMI, a borderline significant loss of lean mass only was found in the appendicular skeletal muscle mass, and not in other body regions (Figure 3). The lean mass, also known as skeletal muscle, is a metabolic organ that generates energy via mitochondrial ATP synthesis [21]. In patients with a high ASMI, the mitochondrial ATP synthesis cycle was functioning well; therefore, the entire body was able to provide the energy needed during CCRT by trading the lean mass. However, in patients with a low ASMI, the mitochondria ATP synthesis cycle might be impaired or energy reservoirs may be depleted throughout the entire body; consequently, there was almost no significant loss of lean mass across the entire body. Thus, it is reasonable to propose that the ASMI could serve as an indicator of mitochondria function; however, future studies should be conducted for further confirmation.

This study had a few limitations. First, computed tomography (CT) is another technique that is used to assess the total body composition of patients with HNC. Pretreatment sarcopenia or subcutaneous adipose tissue detected on CT scans is correlated with survival and treatment-related toxicity in patients with HNC [22,23,24,25]. Although CT is expensive, can evaluate cross-sectional soft tissue, and results in more radiation exposure, it provides a high quality of specificity and accuracy in distinguishing body organs and tissues [26], assessing muscle tissue [27,28], and quantifying visceral and subcutaneous fat tissue [25]. Therefore, future investigations on the relationship between DXA and CT may help shed light on the body composition changes in patients with LAHNC undergoing CCRT. Second, some anthropometric indexes and other body composition analyses have been studied in patients with HNC by using bioelectrical impedance analysis and magnetic resonance imaging [2,11,29]. However, whether the addition of the nutritional assessments to the current model will provide better predictions is unclear. Third, as HNC is not common in women, only three female patients were included in this study. Fourth, muscle function, such as handgrip and gait speed, was not evaluated in this study. Muscle function evaluations are difficult to perform in patients with cancer because port-A or intravenous catheters were placed in most patients and this might cause inaccuracy in measuring muscle function. Lastly, this study is based on limited sample size, single ethnicity and a male-dominant study population.

In conclusion, patients with LAHNC experienced body composition changes and developed a significant loss in BW, BMI, total lean mass, total fat mass, and the ASMI while undergoing CCRT; however, the ASMI was the only independent prognostic predictor of the 2-year RFSR for patients with LAHNC. This might be associated with the asynchronous loss of lean mass in different body regions, and CCRT had a greater influence on the lean mass of peripheral extremities than on the lean mass of the trunk. Thus, in patients with a lower ASMI, there was a lower tolerance for treatment toxicity and they had a significantly lower 2-year RFSR.

## Figures and Tables

**Table 1:** Baseline characteristics of patients with locally advanced head and neck cancer (LAHNC) undergoing concurrent chemoradiotherapy (CCRT).

Variables	Total
	Numbers (%) or Mean ± SD
**Included number of patients**	98 (100)
**Age (years)**	53.4 ± 8.3
**Sex (female:male)**	3 (3.1):95 (96.9)
**Smoking (no:yes)**	6 (6.1):92 (93.9)
**Alcohol consumption (no:yes)**	22 (22.4):76 (77.6)
**Betel nut consumption (no:yes)**	34 (34.7):64 (65.3)
**CCI (0:1:2:** **≥** **3)**	19 (19.4):27 (27.6):19 (19.4):33 (33.6)
**Location**	
**Oral cavity**	50 (51.0)
**Oropharynx**	22 (22.4)
**Hypopharynx**	18 (18.4)
**Larynx**	7 (7.1)
**Unknown primary tumor**	1 (1.0)
**Tumor status (T0:T1:T2:T3:T4)**	1 (1.0):4 (4.1):20 (20.4):10 (10.2):63 (64.3)
**Lymph node status (N0:N1:N2:N3)**	17 (17.3):18 (18.4):57 (58.2):6 (6.1)
**Tumor stage, AJCC 7th edition (III:IVA:IVB)**	9 (9.2):71 (72.4):18 (18.4)
**Treatment modalities**	
**CCRT**	23 (23.5)
**Surgery + CCRT**	49 (50.0)
**Neoadjuvant chemotherapy + CCRT**	26 (26.5)
**Cisplatin dose (mg/m^2^)**	221 ± 57
**RT dose (Gy)**	67.1 ± 4.9
**PG-SGA (well [0–3]:moderate [4–8):severe [≥9])**	74 (75.5):23 (23.5):1 (1.0)
**BMI (kg/m^2^)**	22.9 ± 4.3
**Initial BWL (<10%:** **≥** **10%)**	73 (73.5):26 (26.5)
**2-year RFSR**	57.1%

Abbreviations: LAHNC, locally advanced head and neck cancer; CCRT, concurrent chemoradiotherapy; SD, standard deviation; CCI, Charlson Comorbidity Index; AJCC, American Joint Committee on Cancer; RT, radiotherapy; PG-SGA, patient-generated subjective global assessment; BMI, body mass index; BWL, body weight loss; RFSR, recurrence-free survival rate.

**Table 2:** Univariate and multivariate analyses of prognostic factors associated with the 2-year recurrence-free survival rate (RFSR) of patients with LAHNC.

Variables	Total			
	Univariate Analysis		Multivariate Analysis	
	HR (95% CI)	*p*	HR (95% CI)	*p*
**Age (<60 y vs.** **≥** **60 y)**	1.644 (0.659–4.100)	0.287		
**Sex (female vs. male)**	1.519 (0.133–17.327)	0.737		
**Smoking (no vs. yes)**	1.538 (0.268–8.824)	0.629		
**Alcohol consumption (no vs. yes)**	2.400 (0.848–6.795)	0.099		
**Betel nut consumption (no vs. yes)**	1.964 (0.823–4.687)	0.128		
**CCI (<3 vs.** **≥** **3)**	2.050 (0.876–4.798)	0.098		
**Location (OC vs. NOC)**	0.387 (0.170–0.884)	0.024	0.418 (0.173–1.011)	0.055
**Tumor status (T0–T2 vs. T3–T4)**	2.368 (0.883–6.351)	0.087		
**Lymph node status (N0–N1 vs. N2–N3)**	2.114 (0.887–5.034)	0.091		
**Tumor stage (III vs. IV)**	2.857 (0.562–14.525)	0.206		
**Treatment modalities**				
**CCRT**	1			
**Surgery + CCRT**	0.688 (0.217–2.183)	0.526		
**Neoadjuvant chemotherapy + CCRT**	1.150 (0.424–3.117)	0.784		
**Cisplatin dose (<200 vs.** **≥** **200 mg/m^2^)**	0.681 (0.244–1.902)	0.463		
**RT dose (<68 vs.** **≥** **68 Gy)**	0.769 (0.345–1.716)	0.521		
**PG-SGA (well vs. moderate/severe)**	2.200 (0.882–5.489)	0.091		
**Initial BWL (<10% vs.** **≥** **10%)**	2.831 (1.123–7.138)	0.027		
**Baseline body composition**				
**BMI (<18.5 vs.** **≥** **18.5 kg/m^2^)**	0.245 (0.079–0.764)	0.015	1.412 (0.237–8.418)	0.705
**TLMI (<14.4 vs.** **≥** 14.4 kg/m^2^) *	0.271 (0.102–0.717)	0.009		
**TFMI (<5.1 vs.** **≥** 5.1 kg/m^2^) *	0.391 (0.171–0.895)	0.026	0.519 (0.187–1.443)	0.209
**ASMI (<6.1 vs.** **≥** 6.1 kg/m^2^) *	0.211 (0.083–0.537)	0.001	0.235 (0.062–0.885)	0.030

* ROC curves were used to determine the optimal cutoff value. Abbreviations: RFSR, recurrence-free survival rate; LAHNC, locally advanced head and neck cancer; HR, hazard ratio; CI, confidence interval; CCI, Charlson Comorbidity Index; OC, oral cavity; NOC, non-oral cavity (includes oropharynx, hypopharynx, larynx, and unknown primary); CCRT, concurrent chemoradiotherapy; RT, radiotherapy; PG-SGA, patient-generated subjective global assessment; BWL, body weight loss; BMI, body mass index; TLMI, total lean mass index; TFMI, total fat mass index; ASMI, appendicular skeletal muscle index.

**Table 3:** Body composition changes before and after CCRT in patients with LAHNC.

Overall	Pre-CCRT (*n* = 98)	Post-CCRT (*n* = 98)	Percent Change (95% CI)	*p* Value
BW, kg	63.3 ± 12.3	59.8 ± 10.5	4.9 (−6.5, −3.3)	<0.001
BMI, kg/m^2^	22.9 ± 4.3	21.6 ± 3.7	−4.8 (−6.4, −3.2)	<0.001
TLM, kg	44.0 ± 6.1	41.6 ± 5.5	−5.2 (−6.4, −4.0)	<0.001
TFM, kg	16.3 ± 7.8	15.1 ± 7.0	−5.3 (−9.2, −1.5)	<0.001
TLMI, kg/m^2^	15.9 ± 2.0	15.0 ± 1.8	−5.1 (−6.4, −3.9)	<0.001
TFMI, kg/m^2^	5.9 ± 2.9	5.5 ± 2.5	−4.8 (−9.1, −0.6)	<0.001
ASMI, kg/m^2^	6.8 ± 1.2	6.3 ± 1.1	−7.7 (−9.4, −6.1)	<0.001

Abbreviations: CCRT, concurrent chemoradiotherapy; LAHNC, locally advanced head and neck cancer; CI, confidence interval; BW, body weight; BMI, body mass index; TLM, total lean mass; TFM, total fat mass; TLMI, total lean mass index; TFMI, total fat mass index; ASMI, appendicular skeletal muscle index.

## Data Availability

The data presented in this study are available on request from the corresponding author.

## Supplementary Materials

The following are available online at https://www.mdpi.com/2075-4418/11/2/309/s1.
