# Supplementary material for: Role of the Appendicular Skeletal Muscle Index for Predicting the Recurrence-Free Survival of Head and Neck Cancer
Source: Diagnostics (Basel). 2021 Feb 14;11(2):309. doi: 10.3390/diagnostics11020309 (PMC7918727; doi:10.3390/diagnostics11020309)
Supplement: Supplementary file 1 [file diagnostics-11-00309-s001.pdf]

Supplementary Table S1. Lean muscle changes in the high ASMI group of patients with LAHNC

	**Pre-CCRT (n=69)**	**Post-CCRT (n=69)**	**Percent change (95% CI)**	**P value**
Trunk (kg)	22.5±2.4	21.4±2.4	−4.6% (−6.0, −3.2)	<.001*
Waist (kg)	3.3±0.5	3.1±0.4	−6.0% (−8.0, −4.1)	<.001*
Hips (kg)	6.8±0.9	6.1±0.8	−9.2% (−10.8, −7.6)	<.001*
ASM (kg)	20.4±2.9	18.5±2.8	−9.2% (−10.8, −7.6)	<.001*
Arms (kg)	5.7±0.9	5.1±0.8	−10.4% (−12.0, −8.7)	<.001*
Legs (kg)	14.6±2.1	13.3±2.0	−9.0% (−10.8, −7.2)	<.001*

Abbreviations: ASMI, appendicular skeletal muscle index; LAHNC, locally advanced head and neck cancer; CCRT, concurrent chemoradiotherapy; CI, confidence interval; ASM, appendicular skeletal muscle

Supplementary Table S2. Lean muscle changes in the low ASMI group of patients with LAHNC

	**Pre-CCRT (n=29)**	**Post-CCRT (n=29)**	**Percent change (95% CI)**	**P value**
Trunk (kg)	19.8±1.8	19.6±2.3	−1.2% (−4.1, 1.7)	.403
Waist (kg)	2.9±0.3	2.9±0.4	−1.4% (−5.0, 2.2)	.435
Hips (kg)	5.1±0.6	5.0±0.7	−2.3% (−5.8, 1.2)	.158
ASM (kg)	15.0±1.7	14.3±2.3	−4.2% (−8.4, −0.1)	.049*
Arms (kg)	4.3±0.6	3.9±0.9	−7.4% (−14.6, −0.1)	.057
Legs (kg)	10.6±1.4	10.3±1.8	−2.7% (−6.9, 1.5)	.195

Abbreviations: ASMI, appendicular skeletal muscle index; LAHNC, locally advanced head and neck cancer; CCRT, concurrent chemoradiotherapy; CI, confidence interval; ASM, appendicular skeletal muscle
